# Comparison between transmural and non-transmural infarction and the area at risk using T2 weighted imaging

**DOI:** 10.1186/1532-429X-13-S1-P108

**Published:** 2011-02-02

**Authors:** Tirza Springeling, Alexia Rossi, Adriaan Moelker, Martijn Akkerhuis, Carl Schulz, Evelyn Regar, Piotr Wielopolski, Pim de Feyter, Robert-Jan van Geuns

**Affiliations:** 1 Erasmus MC, Rotterdam, Netherlands

## Introduction

T2 weighted cardiac imaging is currently used as a method to quantify the area at risk (AAR). Recently Ubach et al showed that the AAR exceeds the border of the infarct especially in patients with early reperfusion or aborted myocardial infarction.

## Purpose

In this study we looked at the extend of the salvage area and the relation between transmural, non-transmural infarction and the AAR as well as the myocardial function of the AAR including the infarct, peri-infarct, salvage and remote myocardium**.**

## Methods

The study population consisted of 20 patients with acute ST-elevation myocardial infarction treated by primary PCI. Cardiac MRI was performed within 10 days. The infarct size and the transmural extent of infarction (TEI) and mean TEI was quantified on delayed-enhancement images using 36 segments for each slices. AAR was quantified on T2-weighted images. Transmural infarction was defined as mean TEI>75%. The salvage border was defined as number of segments in AAR without any late enhancement and infarct salvage as AAR minus area with late enhancement. End-diastolic wall thickness (EDWT), segmental wall thickening (SWT) were quantified on cine-images. Myocardial function was calculated in the AAR and included the infarct, peri infarct, salvage border and was compared to remote myocardium

## Results

Infarct mass was 38±23 gram, infarct size was 25±13% of the left ventricle. AAR mass was 70±30 gr, AAR size was 42±15% of the left ventricle. The salvage myocardium was 46±16% of the AAR. Mean TEI was 70±15%. Salvage size is increased in the non-transmural infarct compared to non-transmural infarction (20±7 to 13±7%, p=.02). There was no significant difference in the salvage border between non-transmural and transmural infarction (18±14 and 14±9 gram, p=.43) although the total infarct salvage was increased in the non-transmural infarction (18±10 to 10 ±5 gram, p=.04).

EDWT was increased in AAR compared to the remote myocardium (6.9±0.9 to 6.0±1.0 mm, p=.01), with no difference within the AAR. SWT was impaired in the AAR compared tot the remote (28±17% and 66±18%, p<.01). Within the AAR the SWT was significantly increased in the infarct compared to the salvage myocardium (ANOVA, p<.01).

## Conclusions

The salvage mass extends the borders of the infarction although not significantly different between non-transmural and transmural infarctions. The difference is determined by the salvage of the infarct. In the AAR the EDWT is increased and the SWT is decreased compared to the remote myocardium.

